# Proteome Dynamics and Physiological Responses to Short-Term Salt Stress in *Brassica napus* Leaves

**DOI:** 10.1371/journal.pone.0144808

**Published:** 2015-12-21

**Authors:** Huan Jia, Mingquan Shao, Yongjun He, Rongzhan Guan, Pu Chu, Haidong Jiang

**Affiliations:** 1 State Key Laboratory of Crop Genetics and Germplasm Enhancement, College of Agriculture, Nanjing Agricultural University, Nanjing, China; 2 Jiangsu Collaborative Innovation Center for Modern Crop Production, Nanjing, Jiangsu, China; Northeast Forestry University, CHINA

## Abstract

Salt stress limits plant growth and crop productivity and is an increasing threat to agriculture worldwide. In this study, proteomic and physiological responses of *Brassica napus* leaves under salt stress were investigated. Seedlings under salt treatment showed growth inhibition and photosynthesis reduction. A comparative proteomic analysis of seedling leaves exposed to 200 mM NaCl for 24 h, 48 h and 72 h was conducted. Forty-four protein spots were differentially accumulated upon NaCl treatment and 42 of them were identified, including several novel salt-responsive proteins. To determine the functional roles of these proteins in salt adaptation, their dynamic changes in abundance were analyzed. The results suggested that the up-accumulated proteins, which were associated with protein metabolism, damage repair and defense response, might contribute to the alleviation of the deleterious effect of salt stress on chlorophyll biosynthesis, photosynthesis, energy synthesis and respiration in *Brassica napus* leaves. This study will lead to a better understanding of the molecular basis of salt stress adaptation in *Brassica napus* and provides a basis for genetic engineering of plants with improved salt tolerance in the future.

## Introduction

Salt stress is one of the most severe environmental challenges and limits crop production throughout the world [1]. Research to determine the mechanism of salinity adaptation and improving salt tolerance of plants has attracted increasing attention. NaCl is the main component of salinity [2] and exposure to high concentrations of NaCl can trigger various adverse effects, including water deficit, ionic toxicity, nutritional disorders, plant growth stunt, photosynthesis and protein synthesis depression, excess reactive oxygen species (ROS) generation and oxidative stress [3–6]. To cope with the deleterious effects of salt stress, plants have evolved a series of regulatory mechanisms. Ion homeostasis maintenance, compatible solute accumulation, antioxidant systems, hormonal control, Ca^2+^ signaling and SOS signaling pathways are important in salt-stress tolerance [6,7].

Salt stress can induce extensive proteome alteration in plants and proteomic analysis has proved to be an effective approach to study plant salt-stress tolerance [1,8]. Recently, a database containing 2171 salt-responsive proteins was constructed based on proteomics studies from 34 plant species, which has helped our understanding of the mechanisms underlying plant salt response and tolerance [9]. Two-dimensional polyacrylamide gel electrophoresis (2-DE) remains the primary approach in proteomic research [8] and has been applied commonly in studies on salt stress in plants [10–12].

*Brassica napus* is the third most important source of edible oil in the world and has considerable economic and nutritional values as an oilseed crop [13]. However, *Brassica napus* seedlings are sensitive to salt stress and their growth is markedly inhibited by salinity [14]. Few proteomic studies on the salt-stress response have been reported for *Brassica napus*. A previous study on canola under salt stress detected 44 and 31 differentially accumulated proteins in leaf proteome of the salt-tolerant genotype Hyola 308 and salt-susceptible genotype Sarigol, respectively; 46 proteins were identified using mass spectrometry (MS) analysis [15]. Recently, a proteomic analysis of seedling roots from Hyola 308 and Sarigol detected 20 and 21 proteins that responded to salt-stress treatments, respectively; However, only 19 proteins were identified [16]. The proteome dynamics during salt treatment and the roles of the salt-responsive proteins in salt stress adaptation remain unclear and require further exploration.

To better understand the mechanism of salt-stress adaptation in *Brassica napus*, proteomic and physiological responses to salt stress were analyzed in seedling leaves. Salt-responsive proteins were separated by 2-DE and identified by MS analysis. The functions of these proteins and the dynamic changes in their abundance are investigated and discussed.

## Materials and Methods

### Plant growth and salt treatments

Seeds of *Brassica napus* (var. Nannongyou No.3, salt tolerant) were collected from the Nanjing Agriculture University Agronomy farm in Nanjing city, Jiangsu Province, China. Plants were grown in greenhouse pots filled with a mixture of sand and vermiculite (1:1 v/v) under a light intensity of 120 μmol m^-2^ s^-1^ with a 16 h/8 h photoperiod and temperatures of 23 ± 2°C. The plants were irrigated daily with half-strength Murashige and Skoog solution. Six-week-old seedlings were treated with half-strength Murashige and Skoog solution containing either 200 mM NaCl (salt-stressed treatment group) or no NaCl (unstressed control group) for 24 h, 48 h and 72 h. The third leaves were harvested from control and salt-treated plants, immediately frozen in liquid nitrogen and kept at −80°C until use. At least three independent biological replicates were prepared for physiological and proteomic analyses.

### Biomass and water content

The fresh weight (FW) of *Brassica napus* seedlings and leaves was measured immediately after harvesting. To determine the determination of water content (WC), the plant samples were maintained at 105°C for 5 min and then at 80°C for 72 h in an oven; the dry weight (DW) of the samples was then measured. The WC of the sample was calculated using the following formula: WC = (FW − DW)/FW×100%.

### Pigment determination

Chlorophyll (Chl) was extracted from 0.5 g samples of fresh leaves using 80% acetone. The extract was centrifuged at 12 000 rpm at 4°C for 5 min and the supernatant was collected. Absorbance of the extract was measured at 663 nm and 646 nm spectrophotometrically and the contents of total Chl (Chl a + Chl b), Chl a, and Chl b of leaves were determined as previously described [17]. Three biological replicates were prepared for analysis.

### Leaf gas-exchange measurement

The third leaves of control and salt stressed plants were used for gas-exchange analysis. Net photosynthesis and stomatal conductance were determined by a Li-Cor 6400 portable photosynthesis system (Li-Cor Inc. Lincoln, NE, USA) using the built-in light source set at 1,000 μmol photons m^-2^ s^-1^ [18]. During the measurements, the leaf temperature was adjusted to 25°C.

### Protein extraction and quantification

Protein extraction was performed using the trichloroacetic acid/acetone method, as described previously [19] with some modifications. Leaf samples (1 g) were ground in liquid nitrogen with a mortar and pestle. The tissue powder was transferred to 50 mL centrifuge tubes and suspended in 10% trichloroacetic acid (TCA) and 65 mM DTT in ice-cold acetone and incubated at −20°C overnight. Proteins were pelleted by centrifugation at 40,000 × g for 25 min at 4°C, and then resuspended in cold acetone containing 65mM DTT. The mixture was kept at −20°C for 1 h before centrifugation at 40,000 × g for 15 min at 4°C. Protein pellets were washed twice with cold acetone and lyophilized in a vacuum. The resulting pellet was solubilized in lysis buffer (7 M urea, 2 M thiourea, 4% CHAPS, 65 mM DTT, and 0.2% w/v Bio-Lyte) for 2 h at room temperature and then centrifuged at 16,000 × g for 30 min. The supernatant was collected and stored at −80°C until use. The protein concentration of each sample was measured using the Bradford method [20].

### 2-DE and image analysis

For isoelectric focusing (IEF) in the first dimension, traces of bromophenol blue were added to the protein samples. A total of 450 μg of protein samples was loaded into the immobilized pH gradient (IPG) strips (pH 4–7, 17 cm, Bio-Rad, Hercules, CA, USA). The strips were then covered with mineral oil and rehydrated at 50 V for 14 h at 20°C. Electrophoresis was carried out at 250 V for 1 h, followed by 500 V for 1 h, 1000V for 1h, 2000V for 1h, 10,000 for 4h with a linear ramp and 10,000 V for 80,000 Vh with a rapid ramp. Focused IPG strips were equilibrated as previously described [21] and after equilibration, the strip was placed on the top of 11.5% SDS-PAGE and sealed with 0.5% agarose. Electrophoresis was carried out at 10 mA/gel for 30 min followed by 20 mA/gel until the bromophenol blue dye reached the bottom of the gel. The gels were stained with silver nitrate [22] and analyzed using PDQuest software (version 8.0.1, Bio-Rad, Hercules, CA, USA) for spot detection and protein quantification according to the user manual. Three gel replicates were used for the 2-DE gels quantification. The proteins with at least a two-fold change between the control and treatment samples were considered differentially accumulated proteins (*p* < 0.05 by Student’s *t*-test). Spots with significant changes were selected and manually excised from the gel for further protein identification.

### In-gel digestion and protein identification

The selected spots were in-gel digested with trypsin as previously described [23,24] with minor modification. Briefly, protein spots were washed twice with ultrapure water, destained twice with 25mM NH_4_HCO_3_ in 50% acetonitrile (ACN), dehydrated with 50% ACN and 100% ACN, and then lyophilized in an ALPHA 2–4 LSC Freeze Dryer (Martin Christ, GmbH, Osterode, Germany). The gels pieces were reduced with 10 mM DTT in 25 mM NH_4_HCO_3_ at 56°C for 1 h, alkylated with 50 mM iodoacetamide in 25 mM NH_4_HCO_3_ at 37°C for 30 min, dried with 50% ACN and 100% ACN and digested with trypsin (Promega, Madison, WI, USA) in 25 mM NH_4_HCO_3_ overnight at 37°C. The peptides were extracted twice with 5% trifluoroacetic acid (TFA) in 67% ACN after digestion. All extracts were pooled and lyophilized in the concentrator. The tryptic peptides were prepared by dissolving the extracts with 0.1% TFA and stored at −80°C until MS analysis.

MS analysis was conducted using a matrix-assisted laser desorption ionization time-of-flight (MALDI-TOF/TOF) mass spectrometer 4800 Proteomics Analyzer (AB SCIEX, Framingham, MA, USA). Data were analyzed using GPS 3.6 Explorer software (AB SCIEX, Framingham, MA, USA) and searched using MASCOT 2.1 software (Matrix Science Ltd., London, UK) against the NCBInr database, which contained accessible *Arabidopsis* and *Brassica* protein sequences. The peptide MS and MS/MS tolerances were set as 15 ppm and 0.25 Da, respectively. The search parameters were as follows: specificity of protease digestion was set to trypsin, and a maximum of one missed cleavage was allowed per protein. Carbamidomethyl on Cys was set as a fixed modification and oxidation of Met was set as a variable modification. The probability-based Mowse score was used to determine the confidence of protein identification and only significant hits with a confidence interval (CI) greater than 95% (*p* < 0.05) were accepted as correctly identified.

### Hierarchical cluster analysis

Hierarchical clustering of the relative abundance profiles of differentially accumulated protein was performed on the log2-transformed fold change values of protein spots, using Cluster 3.0 [25]. The complete linkage algorithm was used for data aggregation [21] and TreeView version 1.6 was used to generate a heatmap [18].

### Enzymatic assays

#### Superoxide dismutase (SOD) activity assay

To assess SOD activity, 0.5 g of leaf sample was ground in an ice-chilled mortar with 0.1 M potassium phosphate buffer (pH 7.8) and was centrifuged at 12,000 × g for 20 min at 4°C. The supernatant was used for the enzymatic assay, according to a previously described method [26]. Briefly, 50 μl of the supernatant was added to the 100 mM phosphate buffer (pH 7.8) containing 1.3 μM riboflavin, 13 mM methionine and 65 μM nitro blue tetrazolium (NBT). After the reaction mixture was illuminated for 15 min with luminescent lamps, absorbance was measured at 560 nm. One unit of SOD was defined as the amount of enzyme that induced 50% inhibition of the NBT reduction, and SOD activity was expressed as unit g fresh weight^-1^.

#### Chitinase activity assay

Chitinase activity in leaf samples was measured as previously described [27]. The crude protein extracts were prepared using sodium acetate buffer (pH4.5). After centrifugation at 10,000 × g for 15 min at 4°C, the supernatants were used for the colorimetric determination of N-acetylglucosamine (GlcNAc) with 1% colloidal chitin (w/v) as the substrate. Chitinase activity was calculated from a GlcNAc (Funakoshi, Tokyo, Japan) standard curve. One unit of chitinase activity was defined as the release of 1 μmol of GlcNAc per hour at 37°C.

#### ATPase activity assay

ATPase activity in leaf samples was measured as previously described with modifications [28]. Briefly, 1 g leaf sample was ground in an ice-chilled mortar with 0.05 M potassium phosphate buffer (pH 7.8) containing 0.4 M sucrose and 0.01M NaCl. The supernatants were collected by centrifugation at 1500 × g for 5 min and the pellets were resuspended in 0.05 M potassium phosphate buffer (pH 7.8), 0.4 M sucrose and 0.01 M NaCl at a Chl concentration of 0.5 mg ml^−1^. ATPase activity was determined in a reaction mixture that contained 50 mM Tris-HCl (pH 8.0), 5 mM ATP, and 5 mM CaCl_2_. After incubation at 37°C for 10 min, 20% trichloroacetic acid was added into the mixture to stop the reaction and the concentration of inorganic phosphate (Pi) was determined as described previously [29].

## Results

### Effect of salt stress on plant growth

**Fig 1:**
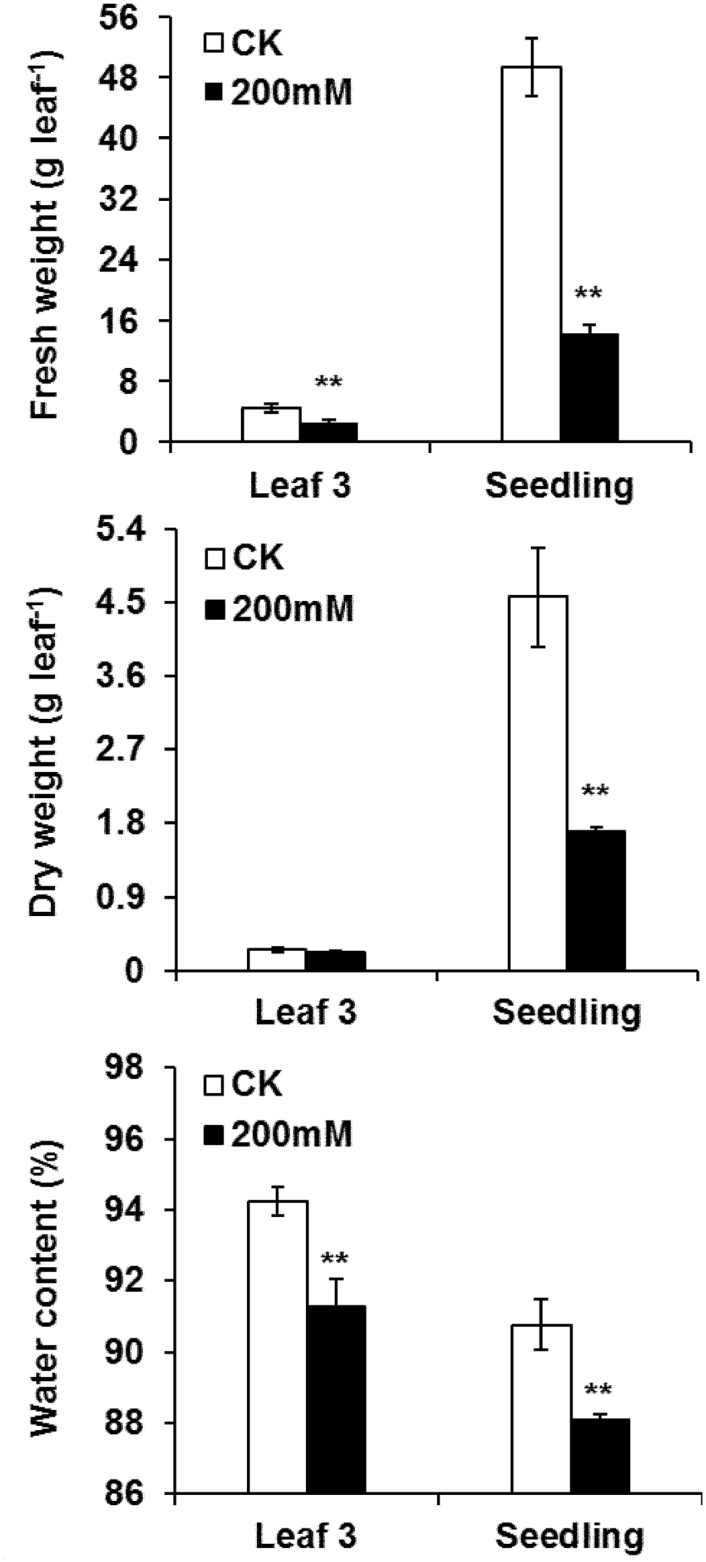
Effects of salt-stress treatments on plant biomass in *B*. *napus* ‘Nannongyou No. 3’. Values shown are means ± SD from three biological replicates. ** indicates a significant difference (compared with the control) at *p* < 0.01.

Salt stress with 200 mM NaCl significantly (*p* < 0.01) decreased the FW, DW and WC of the *Brassica napus* ‘Nannongyou No.3’ seedlings (Fig 1). We further analyzed the effect of salinity on *Brassica napus* leaves, and the results showed that compared with the control plants, the FW and WC of the third leaves was reduced by 43% and 3%, respectively, after salt treatment for 3 d. However, the effect of salinity on the DW of the third leaves was not statistically significant (Fig 1).

### Effect of salt stress on pigments and photosynthesis

**Fig 2:**
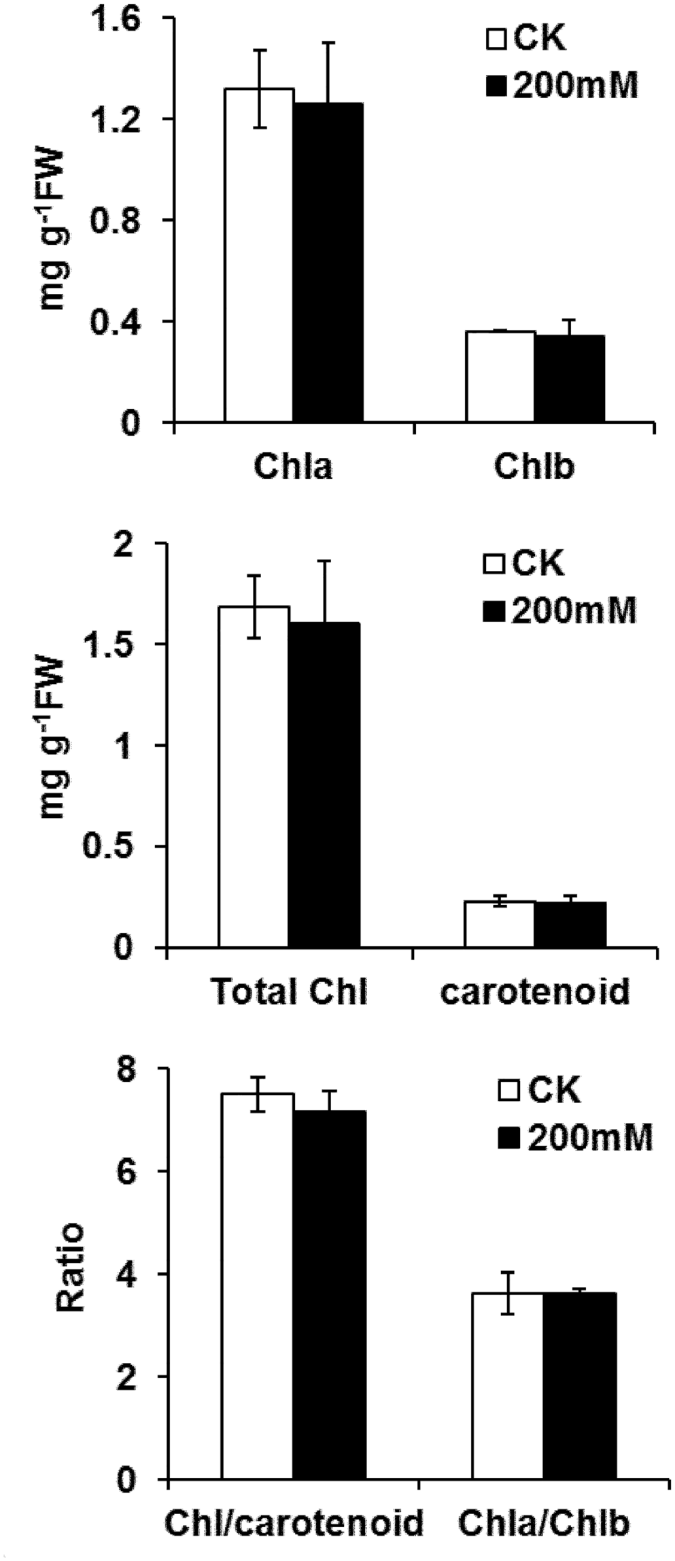
Effect of salt-stress treatments on pigments in *B*. *napus* ‘Nannongyou No. 3’. Values shown are means ± SD from three biological replicates.

No significant decrease in chlorophyll a (Chl a), chlorophyll a (Chl b), total chlorophyll and carotenoid concentrations was observed in the leaves of salt-stressed ‘Nannongyou No.3’ seedlings after salt treatment for 3 d, compared with untreated controls (Fig 2). No significant changes were detected in the Chla to Chlb ratio, and the chlorophyll to carotenoid ratio after salt-stress treatment (Fig 2).

Leaf gas-exchange measurements revealed that net photosynthesis (*Pn*) was dramatically reduced by salinity (Table 1). Salt-stress treatment significantly decreased the stomatal conductance (*Gs*), intercellular CO_2_ concentration (*Ci*) and transpiration rate (*Tr*) by 84%, 27% and 80%, respectively.

**Table 1:** Effects of salt stress on leaf gas exchange in *Brassica napus*.

Treatment	Net photosynthetic rate (μmol CO_2_ m^-2^ s^-1^)	Stomatal conductance (mol H_2_O m^-2^ s^-1^)	Intercellular CO_2_ concentration (μmol CO_2_ mol^-1^)	Transpiration rate (mmol H_2_O m^-2^ s^-1^)
CK	8.62±0.80	0.16±0.016	307.3±6.1	2.75±0.25
200mM	2.81±0.04**	0.03±0.005**	224.7±35.3*	0.54±0.10**

Values shown are means ± SD from three biological replicates:

**: *p* < 0.01;

*: *p* < 0.05.

### Leaf proteome dynamics in response to salt stress

**Fig 3:**
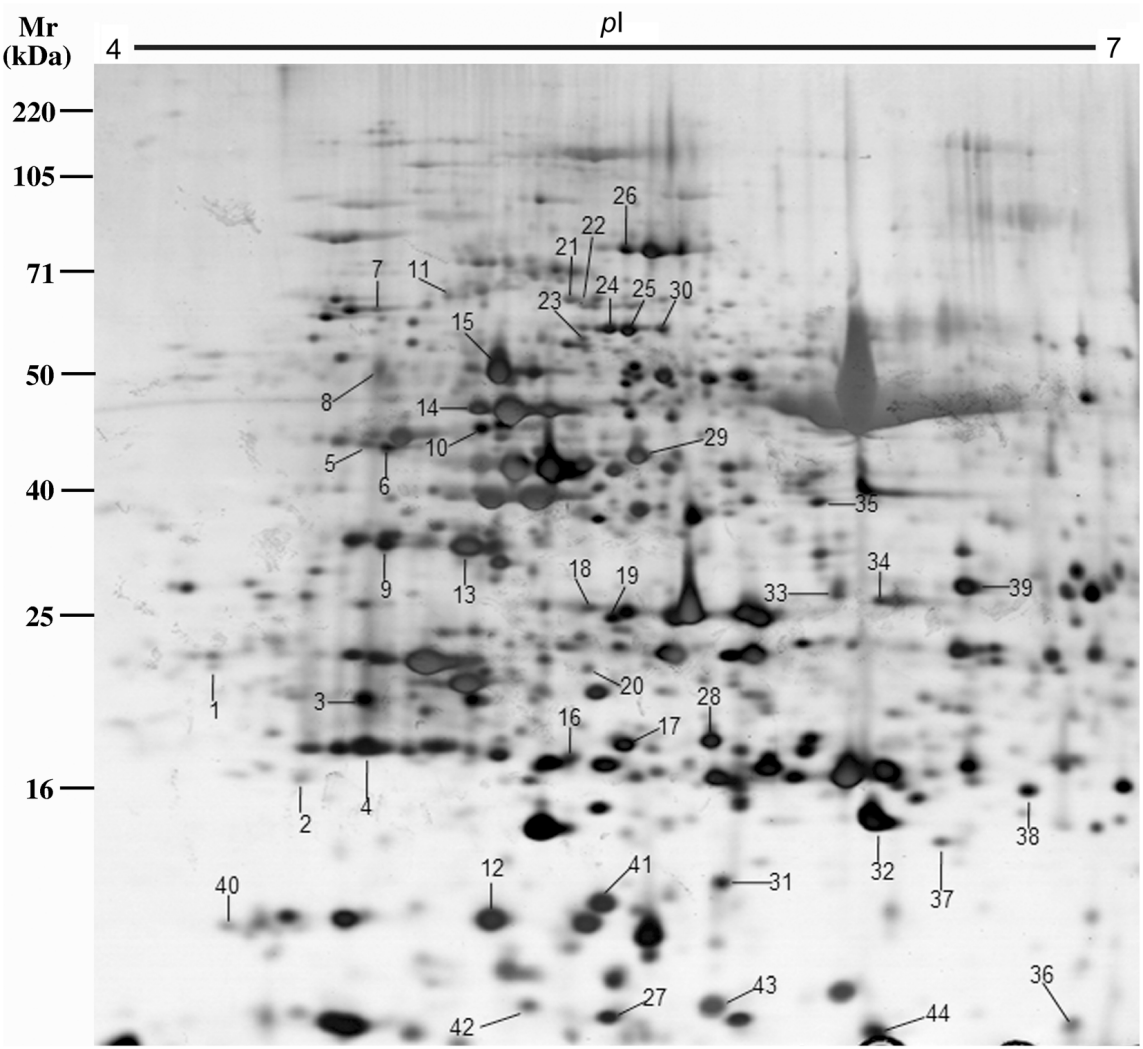
Two-dimensional electrophoresis (2-DE) gel images of proteins extracted from leaf 3 of *B*. *napus* ‘Nannongyou No. 3’ treated with 200 mM NaCl. The 42 salt-stress-responsive protein spots identified by tandem mass spectrometry (MS/MS), which are numbered and indicated by arrows, correspond to the spot numbers in Table 2.

The effects of salt stress on the leaf protein profiles of ‘Nannongyou No.3’ were analyzed using 2-DE. To analyze proteome dynamics in response to salt stress, we measured several time points, from 24 to 72 h, after 200 mM NaCl treatment (S1 Fig). Approximately 800 protein spots were detected on silver-stained 2-DE gels in the pH range of 4–7 by the PDQuest software (Fig 3). Differential protein abundance between the control and salt stressed plants under different NaCl treatment time points was assessed. Forty-four protein spots were observed to be reproducibly and significantly (*p* < 0.05) altered in abundance by more than two-fold in at least one time point after salt treatment. Among these 44 spots, 42 were identified by MS/MS analysis (Table 1, S1 Table and S1 Dataset). ATP synthase (chloroplast) was identified in seven spots (spot 13, 18, 28, 31, 32, 36 and 37), and 60-kDa chaperonin was identified in three spots (spot 4, 16, and 19), whereas chloroplast ribulose-1, 5-bisphosphate carboxylase/oxygenase activase (spot 7 and 30), heat shock protein 70 (spot 20 and 25), and chitinase (spot 26 and 29) were identified in two spots. These phenomena are commonly observed in 2-DE gel analysis and may result from the presence of different isoforms, differential post-translational modification or degradation in the protein extracts; they can also be related to allelic polymorphism [30–33].

**Fig 4:**
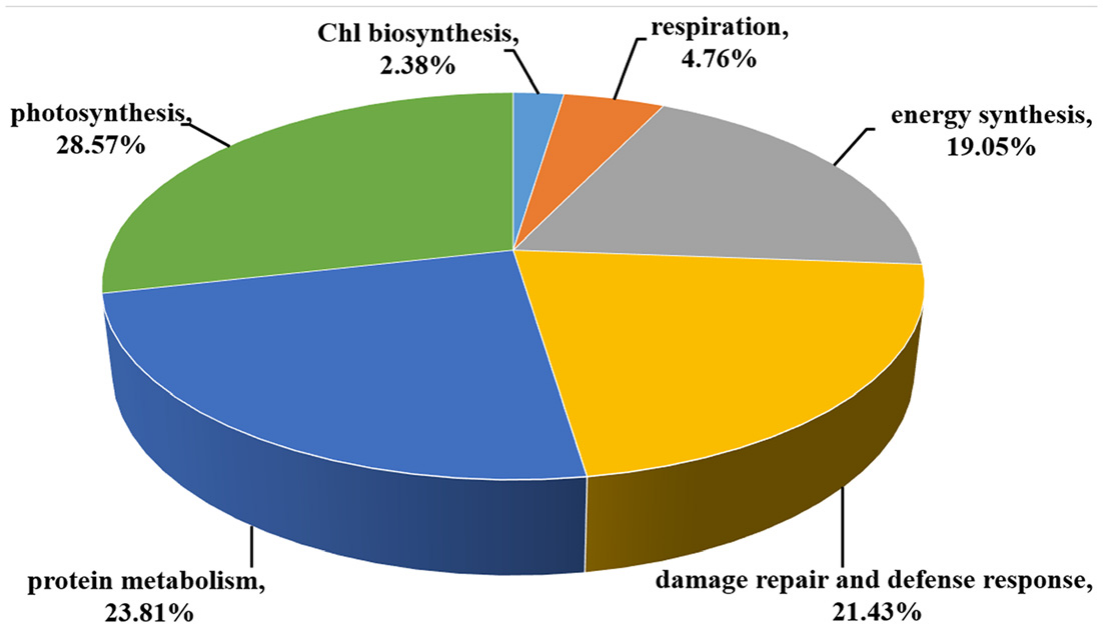
Functional classification of differentially accumulated proteins identified in *B*. *napus* leaves under salt stress.

The identified salt-stress-responsive proteins were further divided into seven categories on the basis of their functions (Fig 4), including Chl biosynthesis (2.38%), photosynthesis (28.57%), energy synthesis (19.05%), respiration (4.76%), protein metabolism (23.81%), and damage repair and defense response (21.43%). At the 24-h time point, 12 protein spots exhibited increased abundance and 16 spots exhibited decreased abundance. The number of down-accumulated proteins upon salt stress decreased and was much lower than the number of salt-induced proteins. Only five spots were down-accumulated, while 15 protein spots were up-accumulated, at the 48-h time point. The changes persisted and at the 72-h time point, the abundance of 18 protein spots was increased and only four spots showed decreases in abundance.

**Fig 5:**
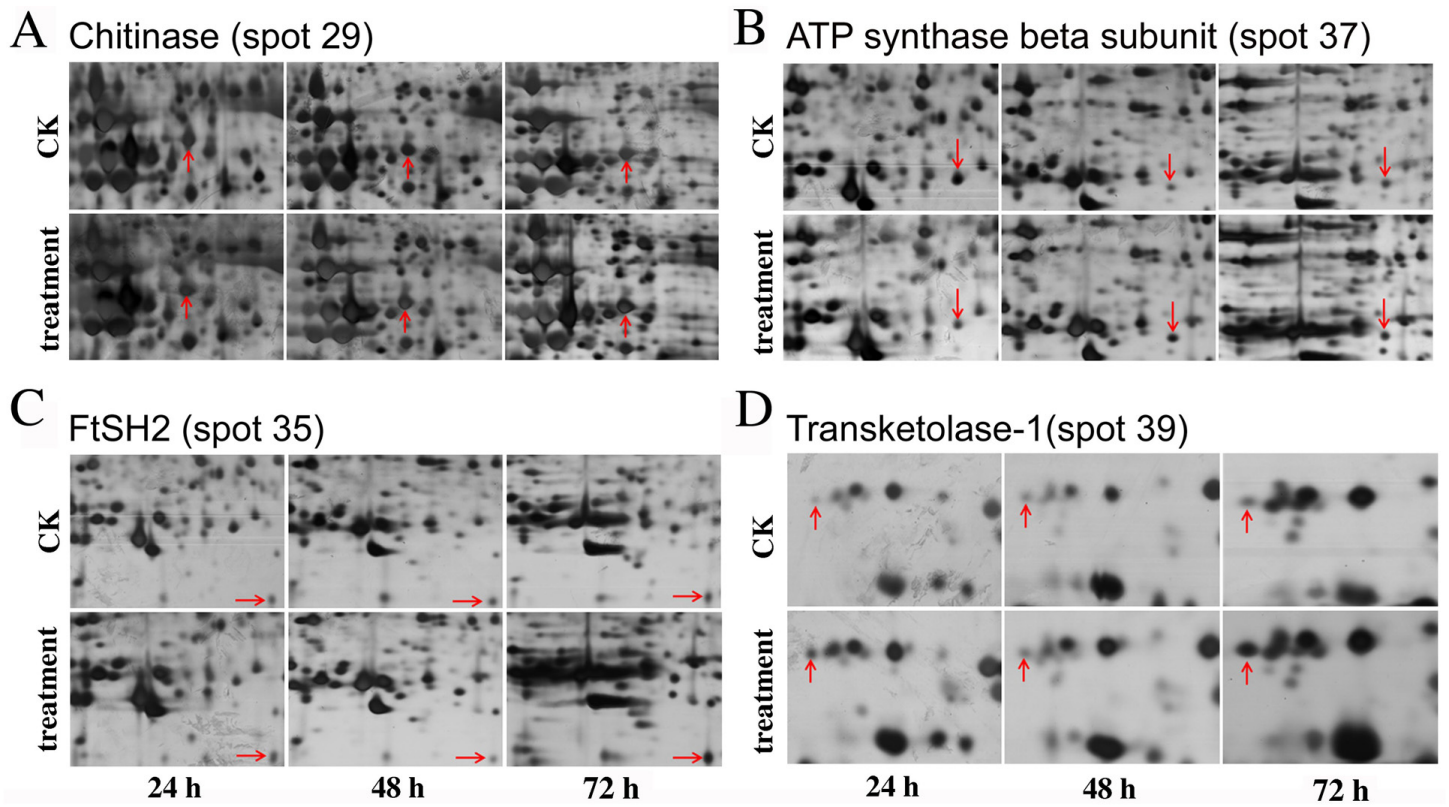
Dynamic patterns of randomly selected salt-responsive proteins at 24 h, 48 h, and 72 h after 200 mM NaCl treatment on *B*. *napus* ‘Nannongyou No. 3’ leaves.

**Fig 6:**
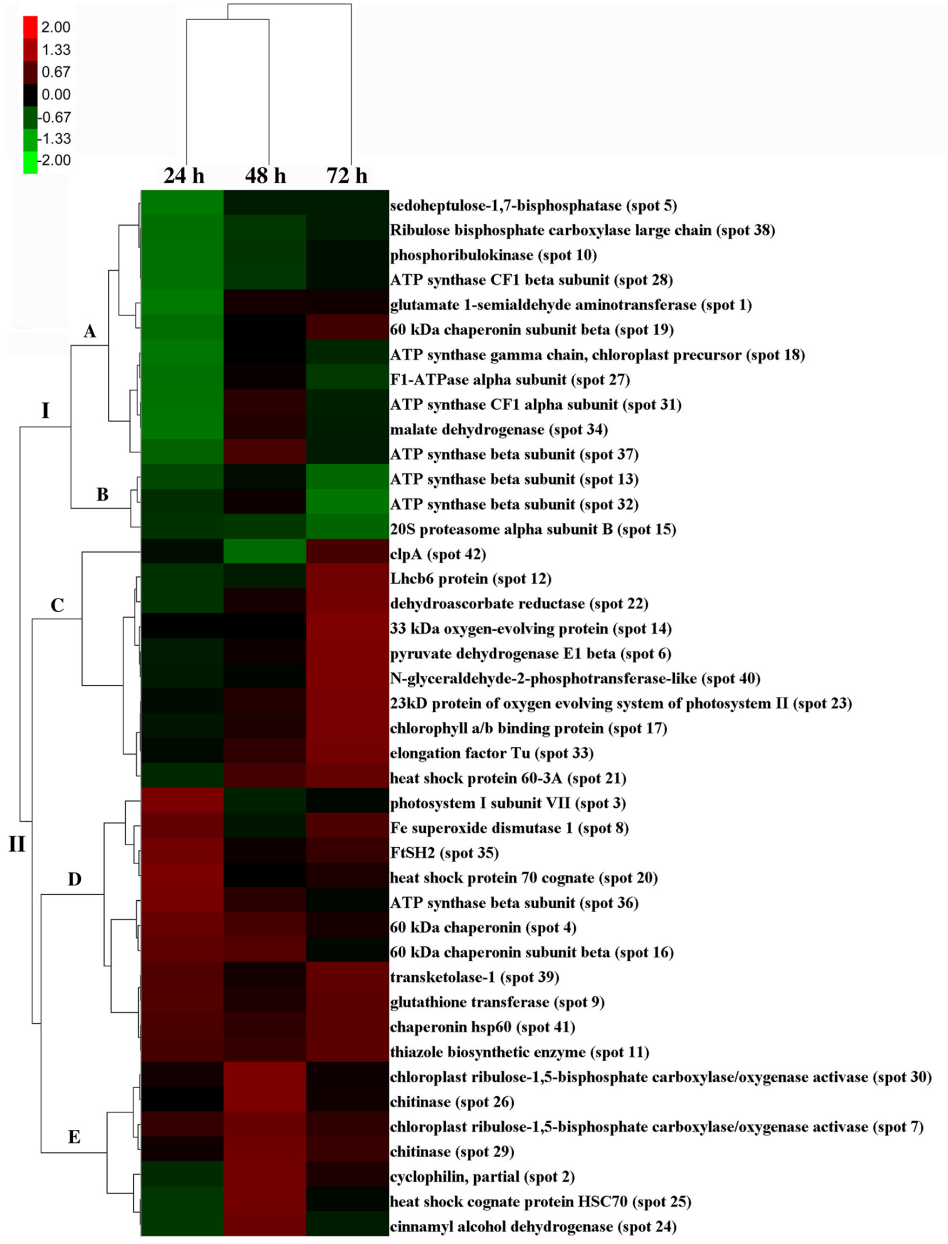
Hierarchical cluster analysis of the dynamic profiles of the 42 identified proteins. Fold changes of protein abundance were log 2 transformed. Columns 1, 2, and 3 represent 200 mM NaCl treatment for 24 h, 48 h, and 72 h, respectively. The rows represent individual proteins. Protein names and spot numbers are labeled to the right of the corresponding heat maps. The proteins that increased and decreased in abundance are indicated in red and green, respectively. Proteins showing no significant changes are indicated in black. The intensity of the colors increases with increasing accumulation differences, as shown to the left of the bar. I and II indicated Cluster I and II, while A-E indicated subgroup A-E.

The salinity-responsive proteins displayed different dynamic patterns during the time course experiments. For example, no significant change in protein abundance was detected for chitinase (spot 29) at 24 h; however, it was up-accumulated at 48 h (Fig 5A). The abundance of the ATP synthase beta subunit (spot 37) was significantly decreased at 24 h, and then increased at 48 h (Fig 5B). However, for FtSH2 (spot 35) and transketolase 1(spot 39), no significant alteration was detected at 48 h, compared with the increased abundance of these two spots at 24 h and 72 h (Fig 5C and 5D). To further reveal the leaf proteome dynamics in response to salt stress, hierarchical cluster analysis was performed (Fig 6). The results indicated that leaves at 24 h and 48 h were grouped into a single group, and leaves at 72 h were in another cluster. The 42 differentially accumulated protein spots in response to salt stress were grouped in two main clusters. The first cluster (Cluster I) included 14 protein spots whose abundance mainly decreased upon high salinity. Most of these proteins are involved in Chl biosynthesis, photosynthesis, respiration and energy synthesis. This cluster could be separated into two subgroups according to the changing patterns of the spots. In subgroup A, the abundance of most down-accumulated proteins at time point 24 h was increased at time point 48 h and 72 h, while the highest depression effect of salinity on the accumulation of proteins in subgroup B was observed at time point 72 h. The second cluster (Cluster II) included 28 protein spots whose abundance was increased by salt treatment. Most of them belong to protein families for protein metabolism, damage repair and defense response. This cluster could be further divided into three subgroups. The first subgroup (subgroup C) included 10 protein spots showing the highest up-regulation upon salt stress treatment at time point 72 h. The accumulation levels of 11 spots in the second subgroup (subgroup D) and seven spots in the third subgroup (subgroup E) was strongly induced by salinity at time point 24 h and 48 h, respectively.

### Changes in enzyme activities in response to salt stress

**Fig 7:**
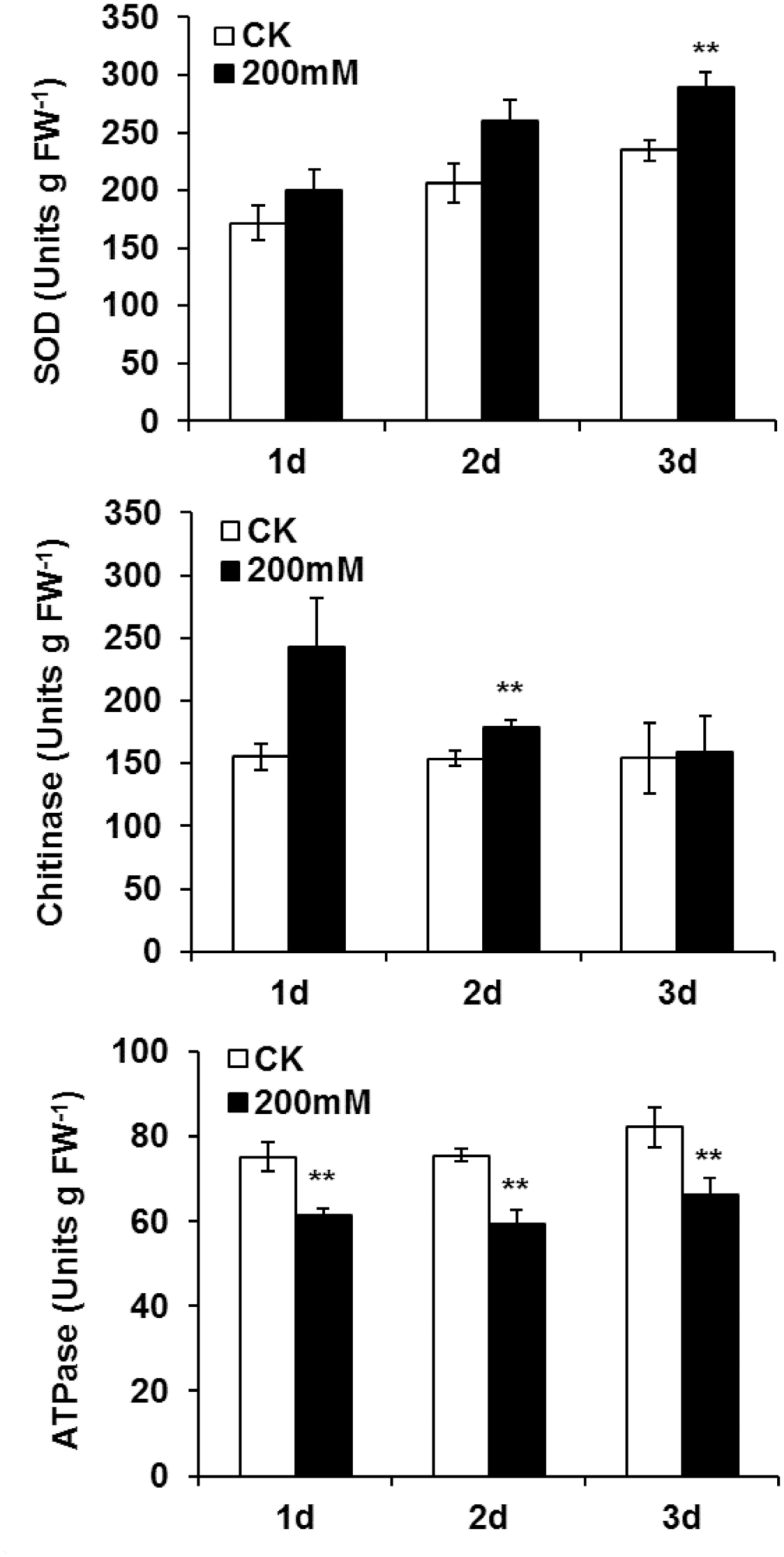
Effects of salt-stress treatments on enzyme activities in leaves of *B*. *napus* ‘Nannongyou No. 3’ seedlings. Values shown are means ± SD from three biological replicates. ** indicates a significant difference (compared with the control) at *p* < 0.01.

The changes in protein abundance identified by 2-DE analysis were further validated by enzyme activity assay. Salt stressed leaves at different time points (24 h, 48 h and 72 h after 200mM NaCl treatment) were tested. In agreement with the data from the proteomics analysis, the activities of SOD and chitinase were significantly increased at 72 h and 48 h of salt stress treatment, respectively, while the ATPase activity was significantly reduced at all three time points (Fig 7).

## Discussion

In this study, the proteomic and physiological responses to salt stress in *Brassica napus* seedling leaves were analyzed. Seedling biomass was reduced after NaCl treatment (Fig 1), implying that plant growth was repressed by salinity. NaCl treatment resulted in a reduction of the *Gs* and *Tr* (Table 1), which might be associated with the water deficiency in salt-stressed leaves. Forty-four salt-stress-responsive proteins were detected, of which 42 were identified (Table 2). The identified proteins were classified into different functional categories. Most of the up-accumulated proteins during NaCl treatment were associated with protein metabolism, damage repair and defense response, which might account for the alleviation of the adverse effect of salt stress on Chl biosynthesis, photosynthesis, energy synthesis and respiration in *Brassica napus* leaves.

**Table 2:** Identification of differentially accumulated proteins under salt stress in leaves of *Brassica napus*.

spot No.^a^	Accession No.^b^	protein name	Species	Mowse Score^c^	Thr.MW/pI^d^	peptide sequence (MS/MS)	cellular location^e^
**chlorophyll biosynthesis**							
1	28972461	glutamate 1-semialdehyde aminotransferase enzyme	*Brassica napus*	62	50562/6.43	DNGALLIFDEVMTGFR	chloroplast
**photosynthesis**							
3	262400776	photosystem I subunit VII	*Brassica napus*	193	9545/6.67	IYDTCIGCTQCVR, CESACPTDFLSVR	chloroplast
5	15228194	Sedoheptulose-1,7-bisphosphatase	*Arabidopsis thaliana*	120	42787/6.17	NEIIRFEETLYGTSR, FEETLYGTSR	chloroplast
7	383470439	Chloroplast ribulose-1,5-bisphosphate carboxylase/oxygenase activase	*Brassica oleracea*	256	48086/6.78	GLAYDTSDDQQDITRGK, LMEYGNMLVMEQENVKR, VQLADQYLNEAALGDANADAIGR	chloroplast
10	15222551	phosphoribulokinase	*Arabidopsis thaliana*	164	44721/5.71	ANDFDLMYEQVK, ILVIEGLHPMFDERVR, KPDFDAFIDPQK	chloroplast
12	50313237	Lhcb6 protein	*Brassica rapa*	27	27324/8.07	TAENFANYTGDQGYPGGR	chloroplast
14	22571	33 kDa oxygen-evolving protein	*Arabidopsis thaliana*	130	32285/5.68	GGSTGYDNAVALPAGGR	chloroplast
17	31323256	chlorophyll a/b binding protein	*Brassica oleracea*	84	29142/5.96	GPSGSPWYGSER, NRELEVIHCR	chloroplast
23	1620920	23kD protein of oxygen evolving system of photosystem II	*Brassica juncea*	139	23448/4.91	WNPSREVEYPGQVLR, EVEYPGQVLR	chloroplast
30	383470439	chloroplast ribulose-1,5-bisphosphate carboxylase/oxygenase activase	*Brassica oleracea*	62	48086/6.78	VQLADQYLNEAALGDANADAIGR	chloroplast
38	1346967	Ribulose bisphosphate carboxylase large chain	*Brassica oleracea*	54	53436/5.88	DTDILAAFR	chloroplast
39	15983513	transketolase-1		124	50553/6.03	SIITGELPAGWEK, ALPTYTPESPGDATR	chloroplast
42	406311	clpA	*Brassica napus*	254	97430/5.90	QLGHNYIGSEHLLLGLLR, TAIAEGLAQR, YRGEFEER, GELQCIGATTLDEYRK, YTDESLVAAAQLSYQYISDR, HAQVPEEARELEK, RPYTVVLFDEIEK	chloroplast
**energy synthesis**							
13	8745523	ATP synthase beta subunit	*Brassica napus*	42	53740/5.21	GMDVVDMGNPLSVPVGGATLGR	chloroplast
18	5708095	ATP synthase gamma chain, chloroplast precursor	*Arabidopsis thaliana*	43	33475/6.12	RPYIPVDKYLEAGTLPTAK, GIYPAVDPLDSTSTMLQPR	chloroplast
27	19554	F1-ATPase alpha subunit	*Brassica napus*	100	55393/6.23	AVDSLVPIGR, EAFPGDVFYLHSR	mitochondrion
28	262400757	ATP synthase CF1 beta subunit	*Brassica napus*	158	53771/5.14	TREGNDLYMEMK, MPSAVGYQPTLSTEMGSLQER, VALVYGQMNEPPGAR	chloroplast
31	262400756	ATP synthase CF1 alpha subunit	*Brassica napus*	167	55325/5.14	IAQIPVSEAYLGR, ASSVAQVVTSLQER, VINALANPIDGR	chloroplast
32	8745523	ATP synthase beta subunit	*Brassica napus*	613	53740/5.21	AHGGVSVFGGVGER, VGLTALTMAEYFR, DVNEQDVLLFIDNIFR, FVQAGSEVSALLGR, ELQDIIAILGLDELSEEDR	chloroplast
36	8745523	ATP synthase beta subunit	*Brassica napus*	204	53740/5.21	AHGGVSVFGGVGER, DVNEQDVLLFIDNIFR, ELQDIIAILGLDELSEEDR	chloroplast
37	8745523	ATP synthase beta subunit	*Brassica napus*	68	53740/5.21	VGLTALTMAEYFR, FVQAGSEVSALLGR	chloroplast
**respiration**							
6	15241286	pyruvate dehydrogenase E1 beta	*Arabidopsis thaliana*	149	39436/5.67	VLAPYSAEDAR, AAEKLAEEGISAEVINLR, IAGADVPMPYAANLER	mitochondrion
34	433335660	malate dehydrogenase	*Brassica oleracea*	126	36038/6.11	ELVKNDEYLNGEFITTVQQR	mitochondrion
**protein metabolism**							
2	167138	cyclophilin, partial	*Brassica napus*	38	18445/8.65	IVMELYADTVPETAENFR	plasma membrane
4	289365	60-kDa chaperonin, partial	*Brassica napus*	104	57714/4.84	NVVLDEFGSPKVVNDGVTIAR, AIELPDAMENAGAALIR	mitochondrion
15	115447473	20S proteasome alpha subunit B	*Oryza sativa*	64	25828/5.39	ETIPVTQLVR	cytoplasm
16	134104	60 kDa chaperonin subunit beta	*Brassica napus*	90	62776/6.56	SAENALYVVEGMQFDR, AAVEEGIVVGGGCTLLR	chloroplast
19	134104	60 kDa chaperonin subunit beta	*Brassica napus*	108	62776/6.56	AAVEEGIVVGGGCTLLR, YEDLMAAGIIDPTKVVR	chloroplast
20	397482	heat shock protein 70 cognate	*Arabidopsis thaliana*	410	71726/5.03	TTPSYVAFTDSER, MVNHFVQEFKR, DAGVIAGLNVMR, IINEPTAAAIAYGLDKK	nucleus
21	18400195	heat shock protein 60-3A	*Arabidopsis thaliana*	67	60771/5.85	ASLDDLAVLTGAEVISEER, ALDNLQTENEDQRR	mitochondrion
25	2655420	heat shock cognate protein HSC70	*Brassica napus*	408	71129/5.07	DAGVIAGLNVMR, IINEPTAAAIAYGLDKK, MVNHFVQEFKR, TTPSYVAFTDSER	nucleus
33	532212746	elongation factor Tu	*Brassica rapa*	411	51394/5.80	KYDEIDAAPEER, QTELPFLLAVEDVFSITGR, VGETVDLVGLRETR, ILDEALAGDNVGLLLR	chloroplast
41	16221	chaperonin hsp60	*Arabidopsis thaliana*	271	61654/5.66	GYTSPYFITNQK, AAVEEGILPGGGVALLYAAR, ELEKLPTANFDQK	cytoplasm
**damage repair and defense response**							
8	312837924	Fe superoxide dismutase 1, partial	*Brassica rapa*	32	22151/5.79	TFMNNLVSWEAVSSR	peroxisome
9	20067415	glutathione transferase	*Triticum aestivum*	78	25098/6.35	KIPVLLHDGR	peroxisome
22	22653413	dehydroascorbate reductase	*Brassica juncea*	203	28687/8.29	ESFKNTEAQTEDVIAGWRPK, NTEAQTEDVIAGWRPK	mitochondrion
11	15239735	thiazole biosynthetic enzyme	*Arabidopsis thaliana*	175	36755/5.82	ALDMNTAEDAIVR, EVVPGMIVTGMEVAEIDGAPR	chloroplast
35	15010596	FtSH2	*Arabidopsis thaliana*	195	36261/4.98	ADILDSALLRPGRFDR, TPGFSGADLANLLNEAAILAGR, AILSEFTEIPPENR	chloroplast
24	19849246	cinnamyl alcohol dehydrogenase	*Lolium perenne*	78	43750/8.55	AGDTVGVGYFLDSCR	cytoplasm
26	6048743	chitinase	*Brassica juncea*	27	44171/4.84	LPGYGVITNIINGGLECAGR	cytoplasm
29	6048743	chitinase	*Brassica juncea*	189	44171/4.84	SFPSFGNTGDLAMR, DLGLELLKNPDVASSDPVIAFK, LPGYGVITNIINGGLECAGR	cytoplasm
40	8885622	N-glyceraldehyde-2-phosphotransferase-like	*Arabidopsis thaliana*	120	31998/5.14	GDKLIEGVPETLDMLR, ENPGCLFIATNR	cytoplasm

^a^ Numbering corresponds to the 2-DE gel in Fig 3.

^b^ Accession number in NCBI database

^c^ Statistical probability of true positive identification of the predicted protein calculated by MASCOT (*p* < 0.05 searching against NCBInr)

^d^ Theoretical molecular weight (kDa) / isoelectrical point (*p*I) of identified proteins

^e^ The cellular location of identified proteins as predicted by TargetP (http://www.cbs.dtu.dk/services/TargetP) and/or PSORT (http://psort.hgc.jp/).

### Chl biosynthesis and photosynthesis under salt stress

Salt stress can impair photosynthesis indirectly by reducing Chl content [6]. However, the impact of salinity on Chl biosynthesis remains unclear. A recent study on rice seedlings suggested that downregulation of Chl biosynthesis by salt stress could be attributed to decreased activities of Chl biosynthetic pathway enzymes [34]. Glutamate 1-semialdehyde aminotransferase (GSA-AT) is a key enzyme in plant Chl synthesis [35], and GSA-AT antisense transformants showed varying degrees of Chl deficiency [36,37]. In this study, the abundance of GSA-AT (spot 1) decreased after 24 h of salt-stress treatment. However, the GSA-AT protein level recovered to control levels at 48 h and 72 h of NaCl treatment (Fig 6), which might be attributed to salt tolerance mechanisms in *Brassica napus* seedlings. Consistent with this finding, the reduction in Chl content in salt-stressed seedlings was not significant after 72 h of treatment (Fig 2).

Photosynthesis is one of the primary processes affected by salinity [38,39]. The decrease in photosynthesis capacity in salt-stressed plants might result from damage to the photosynthetic apparatus and decreased CO_2_ availability caused by stomatal limitations [40,41], other than by reduction of the Chl content. In this study, leaf gas-exchange measurements revealed that net photosynthesis was significantly reduced in salt-stressed seedlings (Table 1). Proteomic analysis showed that 12 proteins related to photosynthesis were salinity responsive, including six photosynthetic proteins and six enzymes crucial for CO_2_ fixation (Table 2). Interestingly, most of the proteins involved in the light reaction, including photosystem I subunit VII (spot 3), Lhcb6 protein (spot 12), 33 kDa oxygen-evolving protein (spot 14), Chl a/b binding protein (spot 17), and 23kD protein of oxygen evolving system of photosystem II (spot 23), were up-accumulated in response to salt stress. However, the protein abundance of enzymes in CO_2_ assimilation, including Ribulose bisphosphate carboxylase (RuBisCO) large chain (spot 38), sedoheptulose-1, 7-bisphosphatase (spot 5) and phosphoribulokinase (spot 10), was significantly decreased after salt-stress treatment, especially at the 24-h time point. In support of this finding, the reduction in stomatal conductance and transpiration rate was far larger than the reduction in intercellular CO_2_ concentration after salt stress treatment (Table 1). These observations suggested that the salt-induced reduction in photosynthesis capacity could be mainly attributed to impaired CO_2_ fixation in *Brassica napus* seedlings. Photosynthesis limited by a reduction in the rate of CO_2_ assimilation by salinity has been reported in wheat [11], rice [42] and Arabidopsis [43].

Two spots (spot 7, 30) corresponding to RuBisCO activase (RCA) were significantly up-accumulated in *Brassica napus* seedlings after salt stress treatment, especially at the 48 h time point. RCA is a key regulatory enzyme that catalyzes the activation of RuBisCO [41]. RCA has been implicated in the maintenance of CO_2_ assimilation at low CO_2_ levels because of the reduction in stomatal conductance caused by salinity [44,45]. The induction of RCA by salinity has been reported previously [15,46] and might explain the recovery of the abundance of RuBisCO at the 48-h and 72-h time point of salt-stress treatment. Therefore, the up-accumulation of RCA might contribute to salt tolerance in *Brassica napus* seedlings.

### Energy synthesis and respiration under salt stress

ATP synthase is a salt-responsive enzyme and plays crucial roles in plant salt tolerance [22,47]. In this study, proteomic analysis identified eight spots (spots 13, 18, 27, 28, 31, 32, 36, 37) as ATP synthase subunits, whose abundances changed under salt-stress conditions. Most of these proteins were down-accumulated, especially at the 24 h time point, and the enzyme activity analysis result was consistent with this finding (Fig 7). These results suggested that energy synthesis might be repressed in salt-stressed *Brassica napus* seedlings. In agreement with this observation, the downregulation of ATP synthase by salt stress has been reported in soybean leaves [48]. However, the response of ATP synthase to salinity depends on the plant species and genotype. For example, the studies in rice [49] and black locust [50] showed that ATP synthase was induced upon salinity. ATP synthase was significantly down-regulated in a salt tolerant cowpea cultivar, but up-regulated in a salt-sensitive cowpea cultivar after salt treatment [44].

Plant respiration response to salt stress has been studied [51]. However, the regulation of respiration by salinity stress appears to be complex, depending on species, tissue or cell type, and stress levels [4,32]. In this study, the abundance of the respiration enzyme pyruvate dehydrogenase (spot 6) was 2.2-fold higher after 72 h of salt stress treatment, while malate dehydrogenase (spot 34) was decreased by 0.66-fold at the 24 h time point. The pyruvate dehydrogenase in mung bean seedlings was reduced in 50 mM NaCl but increased in 100 mM and 150 mM NaCl, while the activity of malate dehydrogenase was decreased in salt-stressed seedlings [52]. The up-regulation of pyruvate dehydrogenase and malate dehydrogenase was reported in tomato leaves under salt stress treatment, especially in a salt-tolerant genotype [53].

### Protein metabolism under salt stress

Salt stress can severely affect protein synthesis [54] and disrupt protein folding in the endoplasmic reticulum (ER), leading to the accumulation of unfolded proteins and ER stress in plants [55]. Proteomic analysis in the present study indicated that elongation factor Tu (spot 33), which is involved in protein biosynthesis and chaperones (cyclophilin, 60 kDa chaperonin/HSP60 and HSP70) related to protein folding, was significantly induced in salt-stressed leaves. By contrast, a protein associated with protein degradation (20S proteasome) was decreased by salinity stress treatment.

The up-accumulation of elongation factor Tu might enhance protein biosynthesis and thus repair the damage of salt stress on photosynthetic proteins in chloroplasts. Similar observations have been reported in cucumber [21] and soybean seedlings [12]. Chaperonins assist protein folding and assembly [56], and may function to protect and repair vulnerable protein targets under stress conditions [33,57,58]. In contrast to our findings, a previous proteomic analysis of *Brassica napus* seedling roots identified chaperonin hsp60 as a down-regulated protein in response to salinity [16]. This differential response could reflect the varying plant genotypes or different tissues used. Cyclophilins are implicated in protection against multiple abiotic stresses, and have been identified as salt-stress-responsive proteins in rapeseed [15], barley [19] and creeping bentgrass [59], consistent with our results. The results suggested that these proteins related to protein metabolism might participate in salt-stress tolerance in *Brassica napus* seedlings.

### Damage repair and defense response under salt stress

Oxidative stress caused by the generation of excess ROS is a well-documented indirect form of damage caused by salt stress [60]. The up-accumulation of antioxidant enzymes, such as FeSOD 1 (spot 8), glutathione transferase (spot 9), and dehydroascorbate reductase (spot 22), might promote ROS scavenging and mitigate the oxidative damage under salt stress in *Brassica napus* leaves. The thiazole biosynthetic enzyme (THI) protein (spot 11) plays a role in thiamine biosynthesis, and its involvement in DNA damage repair and stress-tolerance mechanisms has been observed in bacteria, yeast and *Arabidopsis thaliana* [61–63]. The up-accumulation of THI might help to restore DNA stability and alleviate oxidative stress caused by NaCl treatment in *Brassica napus* leaves. Cell division protein FtSH was suggested to play a role in attenuating the detrimental effects of salinity on the photosynthetic machinery [4], and the enhanced abundance of FtSH2 in salt-stressed *Brassica napus* leaves might have contributed to the restoring of the photosynthetic proteins at 48 h and 72 h after salt-stress treatment (Fig 5).

Three up-accumulated proteins related to the defense response were identified in *Brassica napus* leaves, including cinnamyl alcohol dehydrogenase (spot 24), chitinase (spot 26 and 29) and N-glyceraldehyde-2-phosphotransferase-like protein (spot 40). Cinnamyl alcohol dehydrogenase (CAD) is a key enzyme in lignin biosynthesis and enhanced CAD might increase the extent of lignification, which might represent a salt-adaptation response in plant roots [64–66]. Functional analysis in tea plants suggested that CAD in leaves might play a role in defense against biotic stresses and adaptation to abiotic stresses [67]. However, the function of CAD in salt tolerance in plant leaves is still poorly understood. Chitinase belongs to the pathogenesis-related (PR) protein family, which plays important roles in biotic and abiotic stress resistance [57]. The up-regulation of chitinase by salt stress has been reported in *Brassica rapa* [68], *Nicotiana tabacum* [10] and *Halogeton glomeratus* [69].The increased abundance of chitinase in salt-stressed leaves was further validated and supported by the enzyme assay in the present study (Fig 7). These results suggested that chitinase might help to enhance resistance against salt stress in *Brassica napus*. A study in *Brassica carinata* showed that N-glyceraldehyde-2-phosphotransferase was up-regulated in the resistant genotype upon pathogen attack [70]. The involvement of N-glyceraldehyde-2-phosphotransferase in salt stress resistance/tolerance is unclear, and to the best of our knowledge, it represents a novel salt-stress-responsive protein in *Brassica napus* leaves.

## Conclusions

The present study identified 42 proteins affected by salt stress in *Brassica napus*, including several novel salt stress responsive proteins in plant leaves, such as cinnamyl alcohol dehydrogenase and N-glyceraldehyde-2-phosphotransferase. Proteome dynamics under different time points after NaCl treatment were analyzed. The results suggested that Chl biosynthesis, photosynthesis, respiration and energy synthesis were negatively affected by salt stress at the 24 h time point, and then recovered at the 48 h and 72 h time point. By contrast, protein biosynthesis, damage repair and defense response were significantly induced by NaCl during the stress treatment, implying that proteins in these categories might alleviate the damage caused by NaCl and promote salt adaptation in *Brassica napus*. In this study, analysis of the physiological responses to salt stress supported the proteomic data. This study expanded our knowledge of the mechanisms underlying salt-stress adaptation and will promote molecular breeding of salt-tolerant plants in the future.

## Supporting Information

S1 Dataset The MS/MS spectra for the proteins identified by single peptide. (RAR)

S1 Fig Two-dimensional electrophoresis (2-DE) gel images of proteins extracted from leaf 3 of *B*. *napus* ‘Nannongyou No. 3’. (TIF)

S1 Table Quantification summary of differentially accumulated proteins under salt stress in leaves of *B*. *napus*. Fold change is expressed as the intensity ratio of treatment/control protein spot. Values are presented as means ± SD of the replicates.
(XLSX)
