# Supplementary figures and images for: Proteome Dynamics and Physiological Responses to Short-Term Salt Stress in Brassica napus Leaves
Source: PLoS One. 2015 Dec 21;10(12):e0144808. doi: 10.1371/journal.pone.0144808 (PMC4686907; doi:10.1371/journal.pone.0144808)

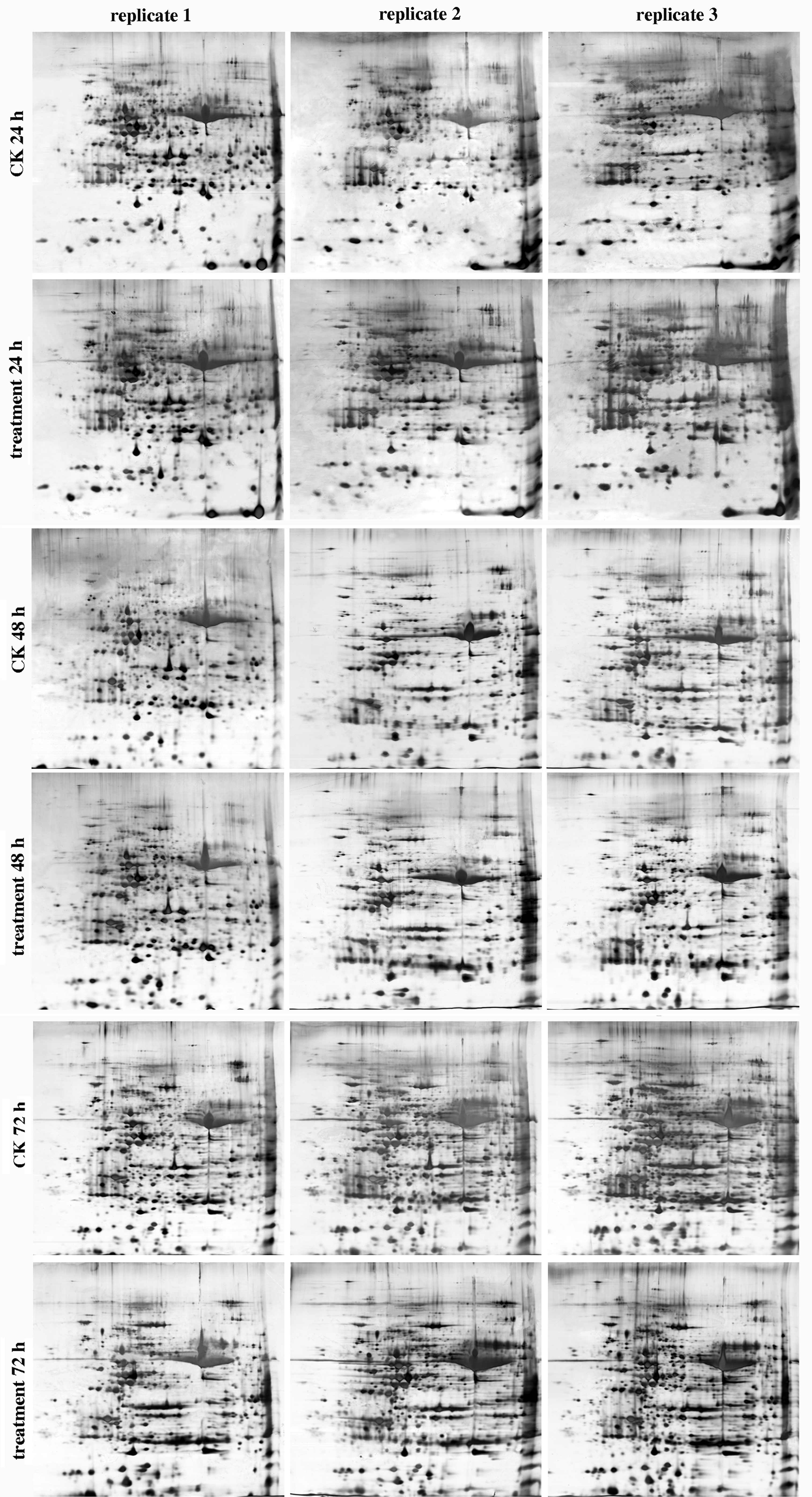

Supplement: S1 Fig — (TIF) [file pone.0144808.s002.tif]
